# West Nile Virus Infections Projected from Blood Donor Screening Data, United States, 2003

**DOI:** 10.3201/eid1203.051287

**Published:** 2006-03

**Authors:** Michael P. Busch, David J. Wright, Brian Custer, Leslie H. Tobler, Susan L. Stramer, Steven H. Kleinman, Harry E. Prince, Celso Bianco, Gregory Foster, Lyle R. Petersen, George Nemo, Simone A. Glynn

**Affiliations:** * Blood Systems Research Institute, San Francisco, California, USA;; † University of California, San Francisco, California, USA;; ‡ Westat, Rockville, Maryland, USA;; § American Red Cross National Testing and Reference Laboratories, Gaithersburg, Maryland, USA;; ¶ University of British Columbia, Victoria, British Columbia, Canada;; # Focus Diagnostics, Cypress, California, USA;; ** America's Blood Centers, Washington DC, USA;; †† Centers for Disease Control and Prevention, Fort Collins, Colorado, USA;; ‡‡ National Heart, Lung, and Blood Institute, Bethesda, Maryland, USA

**Keywords:** West Nile virus, RNA, blood donor, screening, seasonal incidence, research

## Abstract

National blood donor screening for West Nile virus (WNV) RNA using minipool nucleic acid amplification testing (MP-NAT) was implemented in the United States in July 2003. We compiled national NAT yield data and performed WNV immunoglobulin M (IgM) testing in 1 WNV-epidemic region (North Dakota). State-specific MP-NAT yield, antibody seroprevalence, and the average time RNA is detectable by MP-NAT were used to estimate incident infections in 2003. WNV donor screening yielded 944 confirmed viremic donors. MP-NAT yield peaked in August with >0.5% of donations positive for WNV RNA in 4 states. Peak IgM seroprevalence for North Dakota was 5.2% in late September. The average time viremia is detectable by MP-NAT was 6.9 days (95% confidence interval [CI] 3.0–10.7). An estimated 735,000 (95% CI 322,000–1,147,000) infections occurred in 2003, with 256 (95% CI 112–401) infections per neuroinvasive case. In addition to preventing transfusion-transmitted WNV infection, donor screening can serve as a tool to monitor seasonal incidence in the general population.

## Abstract 2

Routine donor nucleic acid amplification testing is a valuable surveillance screening tool.

After its identification in New York City in 1999, West Nile virus (WNV), a mosquitoborne flavivirus, emerged as a cause of neuroinvasive disease (meningitis, encephalitis, and acute flaccid paralysis) and febrile illness in the United States (*1**–**5*). Since 2000, a national surveillance system, ArboNET, has monitored WNV activity in mosquitoes, horses, and other animals, as well as cases of febrile illness and neuroinvasive disease in humans (*2*). Seroprevalence studies after epidemics indicate that febrile illness develops in ≈20% of infected persons, while neuroinvasive disease develops in <1% (*6**,**7*). On the basis of reported neuroinvasive cases and an estimated ratio of the number of infections to neuroinvasive cases, as of October 2004, a total of ≈1 million persons have been infected with WNV in the United States (*2*).

Evidence accumulated in 2002 that WNV could be transmitted by blood transfusion, culminating in 23 documented cases that year (*8**–**10*). In late 2002, the US Food and Drug Administration (FDA), US blood collection organizations, and test manufacturers began an accelerated program to implement nucleic acid amplification testing (NAT) of blood donors for West Nile viremia before the 2003 season (*11**,**12*). Assays were developed for use in a minipool-NAT format (i.e., samples of donations are pooled, and the pool is tested), similar to procedures now routinely used for blood screening for HIV-1 and hepatitis C virus (HCV) by NAT (*13*). In addition to minipool-NAT screening, several blood centers performed individual donation NAT screening in regions experiencing epidemic WNV activity to interdict donations with low-level viremia that could be missed by minipool-NAT (*14**–**18*).

Synthesis of blood donor screening data may provide an opportunity for public health surveillance in addition to ArboNET because of the large number of donations screened from a broad cross-section of the adult population. We report the combined results of WNV donor screening during the summer and fall of 2003 by America's Blood Centers (ABC) and the American Red Cross (ARC), which together collect and test ≈95% of US donations. In addition, WNV IgM and IgG testing was performed on donor specimens from 1 WNV-epidemic region to determine the proportion of donors with measurable antibody responses to WNV during 2003. We used this proportion, along with the minipool-NAT data from that region, to determine the average time during which WNV RNA was detectable by minipool-NAT. This time was combined with the minipool-NAT donor screening data for each state and US Census data to estimate the proportion of WNV-infected persons (seasonal incidence) in each state and the total number of incident infections nationwide in 2003.

## Methods

### Overview of Approach

Since July 2003, all blood donations have been screened for WNV RNA by NAT. If we assume that blood donation and WNV infection are independent events, the proportion of blood donors infected by WNV in 2003 (seasonal incidence of WNV in the blood donor pool) is a function of NAT yield and the average length of time that WNV RNA is detectable after infection. By measuring IgM antibodies in North Dakota blood donors shortly after the epidemic, we estimated the seasonal incidence for that region. After adding NAT screening yield data from the same donor population, we then estimated the length of time that WNV RNA is detectable by NAT.

We combined the length of time that RNA is detectable by NAT with NAT screening yield data by state to estimate state-specific and national WNV seasonal incidence in the blood donor and general population. Finally, by dividing the estimated number of infections in the general population in 2003 by the number of neuroinvasive disease cases reported to the national WNV surveillance system (ArboNET), we estimated the ratio of WNV infections to neuroinvasive disease cases.

### Blood Donor Screening

US blood donations are screened for WNV RNA by using NAT assays on pools of 6 to 16 donations or on individual samples in high-incidence regions. In 2003, ≈96% of the screening was conducted on pooled samples. Additionally, 2 blood collection organizations (ARC; Blood Systems) retrospectively performed individual donation NAT on cryopreserved plasma from 36,269 donations in 5 states with substantial epidemics to ascertain the proportion of low-level viremia missed by minipool-NAT and to assess, through recipient lookback, the infectivity of units harboring low-level viremia (*15**,**16*). Viremic donations detected by individual donation NAT were included in this analysis to compile total NAT yield for 2003, but they were excluded from calculations used to project WNV infection in the general population for the following reasons: 1) inconsistent application of individual donation NAT screening around the country (*15**,**16*); 2) variable rate of detection of low-level viremia by individual donation NAT assays (*18*); and 3) fever and symptoms during the postseroconversion low-level viremia phase (unlike the asymptomatic minipool yield phase), which would result in self-deferral from donation and bias our projections.

### West Nile Viremia in US Blood Donors: Geographic and Temporal Distribution

We combined 2 large databases consisting of all donations and WNV-confirmed donations obtained by state from July 1 (when most blood centers implemented WNV NAT screening) to October 31, 2003. The first database was derived from 72 of the 74 independent blood centers that constitute ABC, which collect nearly 50% of US donations (*14**,**19*). Data elements included total number of donations, donor state of residence, and the minipool and individual donation NAT confirmatory status of all donations collected from July to October 2003. Similar data were obtained from the ARC national donor database, which constitutes 45% of the US supply (*16*). Donations were classified as confirmed WNV NAT-positive if the index donation was reactive by NAT and 1) positive for IgM or by an alternative NAT procedure or 2) follow-up samples from donors were reactive on a NAT assay or were IgM-positive. The dataset included the subset of confirmed NAT-positive donations that were either originally detected by minipool-NAT, or had been detected by prospective individual donation NAT but were subsequently determined to be detectable by minipool-NAT (were reactive when retested at a 1:16 dilution using minipool-NAT). This extra testing ensured that seasonal incidence estimates were based on data obtained by using a comparably sensitive screening process across all regions of the United States and throughout the epidemic period. The proportion of confirmed positive donations identified by minipool-NAT was determined by month for each state, and ≈95% confidence intervals (CIs) around these minipool-NAT yield estimates were computed (*20*). The Epi Map component (Environmental Systems Research Institute, Redlands, CA, USA) of EpiInfo version 3.3 (Centers for Disease Control and Prevention [CDC], Atlanta, GA, USA) was used to display results graphically.

### Estimate of Days West Nile Viremia Is Detectable by Minipool-NAT

To use NAT screening data to estimate state-specific WNV seasonal incidences, we first derived an estimate for the average length of time that RNA is detectable by minipool-NAT after infection occurs (TMP-NAT). TMP-NAT can be approximated if both the minipool-NAT screening yield and seasonal incidence of WNV are known (Appendices 1 and 2; available from http://www.bsrisf.org/eid2006/app1.html and http://www.bsrisf.org/eid2006/app2.html). The seasonal incidence was estimated by measuring the peak WNV IgM prevalence observed in a particular region ≈3 weeks after the end of the region's first epidemic (http://www.bsrisf.org/eid2006/app1.html). Serologic data allowed us to evaluate both minipool-NAT yield and prevalence of IgM for each week from July to September 2003 and to identify peak IgM prevalence. The sum of the weekly minipool-NAT yield estimates divided by the peak IgM prevalence (our estimate of the 2003 seasonal incidence in North Dakota) was used to derive TMP-NAT (http://www.bsrisf.org/eid2006/app2.html). Approximate 95% CIs around peak IgM prevalence and TMP-NAT were calculated by assuming normal distributions with variances approximated by Taylor series (*21*).

### WNV Seasonal Incidence

We assumed that WNV infection dynamics are similar in blood donors and in the general population. The monthly WNV incidence in each state for each month from July through October was derived by multiplying the monthly minipool-NAT yield by the number of days in each month and dividing by the average period of time during which RNA is detectable (TMP-NAT) (Appendix 3; available from http://www.bsrisf.org/eid2006/app3.html). Each state-specific seasonal WNV incidence was calculated by summing the 4 monthly WNV incidence estimates. To estimate 2003 WNV infections nationwide, we multiplied each state-specific seasonal WNV incidence by the corresponding July 1, 2003, population estimate from the US Census Bureau (*22*) and then summed over all states. An ≈95% CI around the estimated 2003 WNV infections nationwide was calculated by assuming a normal distribution with variance approximated by Taylor series (*21*).

### Proportion of West Nile Infections Resulting in Neuroinvasive Disease

We then calculated the ratio of the estimated number of WNV infections nationwide and the total neuroinvasive disease cases reported to CDC (*23*). The standard error (SE) of this ratio is dependent on the SE of the total neuroinvasive disease cases (assumed to be Poisson distributed), the SE of TMP-NAT, and the SEs of state-specific minipool-NAT yield estimates (assumed to be binomially distributed) and was approximated by a Taylor series (*21*). We did not estimate the proportion of infections resulting in West Nile–related febrile illness because it is considerably underreported to ArboNET.

### Approvals for Research on Human Subjects

The Investigational New Drug protocols, which included donor consent for WNV NAT screening and follow-up testing, were reviewed and approved by multiple institutional review boards and FDA. Institutional review board approval of this study protocol, including compilation of a national NAT yield database and anonymous IgM and IgG testing (http://www.bsrisf.org/eid2006/app1.html), was obtained from the University of California, San Francisco Committee for Human Research, and from Westat (Rockville, MD, USA).

## Results

**Figure 1:**
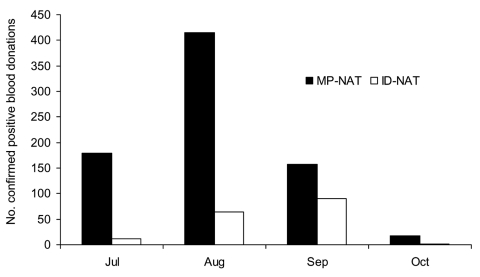
Yield of West Nile virus nucleic acid amplification test (NAT) screening of 4,585,573 donations at American Red Cross and America's Blood Centers (constituting ≈95% of US collections) from July 1 to October 31, 2003. A total of 944 confirmed viremic donations were identified, including 770 that were detectable by minipool-NAT and 174 detectable only by individual donation NAT. MP, minipool; ID, individual donation.

**Figure 2:**
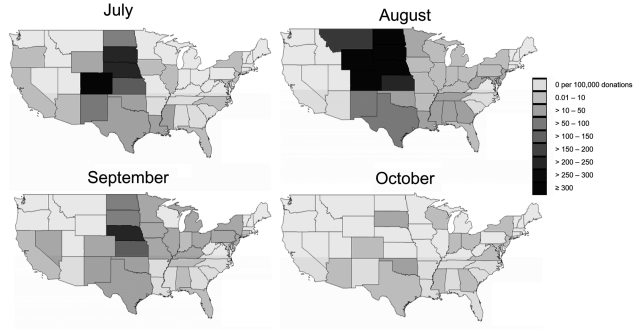
Yield of minipool–nucleic acid testing of blood donors for West Nile virus RNA by state and month, 2003.

Overall, 944 confirmed West Nile viremic donors (0.02%) were identified by NAT screening among 4,585,573 donations from July 1 to October 31, 2003, at ARC and participating centers in ABC. These included 770 donations detected by minipool-NAT and 174 donations detected only as a result of prospective or retrospective individual donation NAT. The distribution of minipool-NAT and individual donation NAT yield by month is shown in Figure 1. Of the 191 viremic donations detected in July, only 2 were detected in the first week of July (both on July 6), and only 4 confirmed viremic donations were reported by ABC or ARC after October 31; thus, the July–October period composes virtually the entire 2003 epidemic. Geographically, the epidemic was most dramatic in the Central Plains states. The rate of WNV-infected donations exceeded 3 per 1,000 in Colorado in July and August and in 4 additional contiguous states in August (Figure 2).

### North Dakota Data

**Figure 3:**
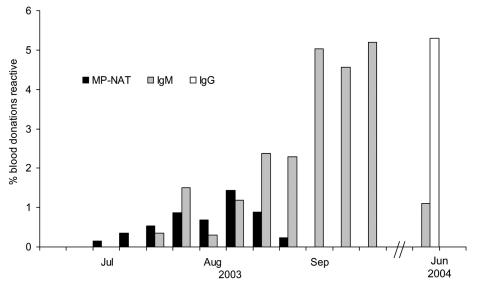
West Nile virus minipool–nucleic acid amplification testing (MP-NAT) yield and immunoglobulin M (IgM) and IgG seroprevalence estimates for North Dakota, during and ≈8 months after the 2003 epidemic period.

As shown in Figure 3, minipool-NAT–confirmed positive donations were detected from July 13 to September 6, 2003, in the Bismarck and Minot regions of North Dakota, with minipool-NAT yield peaking at 1.4% (95% CI 0.4–2.3) in late August. IgM-confirmed positive donations were not observed in these same regions during the first 3 weeks of July but were detected toward the end of July. IgM prevalence gradually increased thereafter and reached a plateau around September 7; ≈5% of donations were positive for IgM during most of September. The peak IgM prevalence was observed the last week of September (5.2%, 95% CI 3.0–7.4) and was similar to the IgG prevalence observed 9 months later in June 2004 (5.3%, 95% CI 3.9%–6.7%), when IgM prevalence had declined to 1.2%. Thus, the peak IgM weekly prevalence was assumed to be a good estimate of the seasonal incidence in this region. The average length of time viremia is detectable by minipool-NAT, TMP-NAT, was estimated to be 6.9 days (95% CI 3.0–10.7).

### WNV Seasonal Incidence

**Figure 4:**
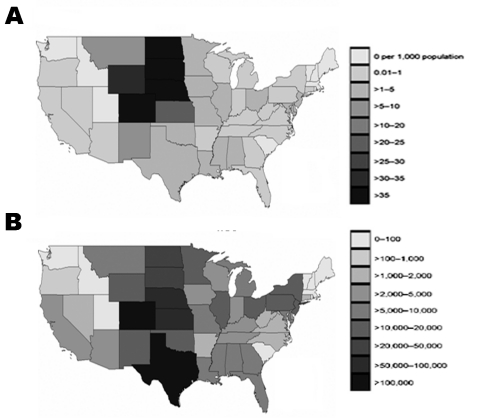
A) Projected number of West Nile virus (WNV) infections per 1,000 persons. B) Estimated total number of WNV infections per state during 2003 epidemic season.

The proportion of the population estimated to have become infected during 2003 in each state was 0%–4.9% (Figure 4A and Table). The highest proportions were observed in Nebraska (4.9%), Colorado (4.3%), North Dakota (4.1%), South Dakota (4.0%), Wyoming (3.5%), and Kansas (2.1%). Nationally, 735,000 persons (95% CI 322,000–1,147,000) were estimated to have been infected in 2003 (Table). Figure 4B shows the distribution of these infections by state. The greatest number of infections were located in Colorado, Texas, Nebraska, Kansas, the Dakotas, and to a lesser extent the states in the Midwest and Northeast, which had only moderate seasonal incidence but have large populations.

**Table:** Estimated WNV seasonal incidence and related measures of WNV infection for each state and entire United States, 2003*

State	No. MP-NAT–positive donations, 4-mo period	No. donations, 4-mo period	Estimated WNV seasonal incidence, % (95% CI)	Midyear population estimate†	Estimated no. infections	Neuroinvasive cases reported to CDC‡
Alabama	3	48,044	0.12 (0.00–0.27)	4,500,752	5,336	25
Alaska	0	8,444	0.00 (0.00–0.00)	648,818	0	0
Arizona	2	86,157	0.04 (0.00–0.10)	5,580,811	2,291	7
Arkansas	1	49,750	0.04 (0.00–0.12)	2,725,714	1,050	23
California	2	394,470	0.01 (0.00–0.02)	35,484,453	3,231	2
Colorado	157	65,739	4.33 (1.83–6.83)	4,550,688	197,028	621
Connecticut	1	52,410	0.03 (0.00–0.10)	3,483,372	1,121	12
Delaware	3	19,853	0.28 (0.00–0.63)	817,491	2,268	12
District of Columbia	0	3,055	0.00 (0.00–0.00)	563,384	0	3
Florida	6	248,198	0.05 (0.00–0.09)	17,019,068	7,677	61
Georgia	6	118,981	0.09 (0.00–0.18)	8,684,715	7,803	27
Hawaii	0	16,981	0.00 (0.00–0.00)	1,257,608	0	0
Idaho	0	23,204	0.00 (0.00–0.00)	1,366,332	0	0
Illinois	15	236,926	0.11 (0.03–0.20)	12,653,544	14,399	30
Indiana	4	111,090	0.06 (0.00–0.14)	6,195,643	3,944	15
Iowa	6	89,649	0.12 (0.00–0.23)	2,944,062	3,476	81
Kansas	70	59,673	2.13 (0.85–3.42)	2,723,507	58,136	89
Kentucky	3	68,674	0.08 (0.00–0.18)	4,117,827	3,298	11
Louisiana	6	56,284	0.19 (0.00–0.38)	4,496,334	8,649	101
Maine	0	24,968	0.00 (0.00–0.00)	1,305,728	0	12
Maryland	7	75,403	0.17 (0.01–0.33)	5,508,909	9,418	49
Massachusetts	0	65,914	0.00 (0.00–0.00)	6,433,422	0	12
Michigan	7	174,776	0.07 (0.01–0.14)	10,079,985	7,122	14
Minnesota	22	114,571	0.35 (0.11–0.59)	5,059,375	17,589	48
Mississippi	6	47,980	0.23 (0.01–0.45)	2,881,281	6,548	34
Missouri	9	124,644	0.13 (0.02–0.24)	5,704,484	7,342	39
Montana	8	19,984	0.76 (0.08–1.43)	917,621	6,956	75
Nebraska	161	61,516	4.87 (2.06–7.68)	1,739,291	84,648	194
Nevada	1	32,651	0.06 (0.00–0.17)	2,241,154	1,267	2
New Hampshire	0	24,595	0.00 (0.00–0.00)	1,287,687	0	2
New Jersey	9	98,008	0.17 (0.02–0.31)	8,638,396	14,269	21
New Mexico	10	25,130	0.73 (0.12–1.34)	1,874,614	13,701	74
New York	10	245,357	0.07 (0.01–0.13)	19,190,115	13,774	57
North Carolina	1	146,807	0.01 (0.00–0.04)	8,407,248	1,087	16
North Dakota	30	13,971	4.14 (1.40–6.88)	633,837	26,264	94
Ohio	13	214,819	0.11 (0.02–0.19)	11,435,798	12,112	84
Oklahoma	23	98,287	0.43 (0.13–0.73)	3,511,532	15,196	56
Oregon	1	72,337	0.03 (0.00–0.08)	3,559,596	966	0
Pennsylvania	14	255,806	0.10 (0.02–0.17)	12,365,455	12,010	145
Rhode Island	0	580	0.00 (0.00–0.00)	1,076,164	0	5
South Carolina	0	70,369	0.00 (0.00–0.00)	4,147,152	0	3
South Dakota	46	20,430	4.02 (1.50–6.54)	764,309	30,716	151
Tennessee	4	88,532	0.08 (0.00–0.17)	5,841,748	4,797	21
Texas	80	309,469	0.48 (0.19–0.77)	22,118,509	106,013	431
Utah	0	35,448	0.00 (0.00–0.00)	2,351,467	0	0
Vermont	0	16,616	0.00 (0.00–0.00)	619,107	0	0
Virginia	1	77,634	0.02 (0.00–0.07)	7,386,330	1,623	19
Washington	0	93,469	0.00 (0.00–0.00)	6,131,445	0	0
West Virginia	1	25,560	0.07 (0.00–0.23)	1,810,354	1,342	1
Wisconsin	4	142,456	0.05 (0.00–0.11)	5,472,299	2,863	7
Wyoming	17	9,904	3.48 (0.94–6.02)	501,242	17,439	92
Total	770	4,585,573	0.25 (0.11–0.39)	290,809,777	735,000 (322,000–1,147,000)§	2,866

*WNV, West Nile virus; MP-NAT, minipool-nucleic acid amplification testing; CI, confidence intervals. †US Census Bureau (*22*). ‡Source: Centers for Disease Control and Prevention (*23*). §Confidence intervals.

### Reported WNV Neuroinvasive Disease Relative to Projected Infection Incidence

**Figure 5:**
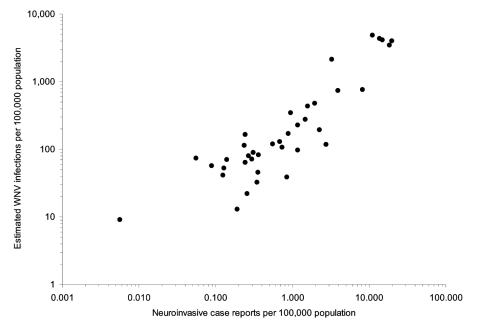
Projected proportion of each state's population infected with West Nile virus versus the proportion of the state's population reporting neuroinvasive disease cases to the Centers for Disease Control and Prevention's ArboNET program. Data are excluded for 13 states: 6 states with neither minipool–nucleic acid amplification testing (MP-NAP) yield nor neuroinvasive cases, 6 states with 2 to 12 neuroinvasive cases but no MP-NAT yield, and 1 state with 1 MP-NAT–positive donor but no reported neuroinvasive cases.

We compared the estimated proportion of the population infected with WNV to the proportion of WNV neuroinvasive disease cases reported to CDC for each state. Figure 5 shows that these proportions are highly correlated with one another. A total of 2,866 neuroinvasive WNV cases were reported nationally to CDC's ArboNET system in 2003 (Table). This total was compared to 735,000 persons nationally estimated to have been infected with WNV in 2003. Thus, an estimated 256 WNV incident infections occurred per reported neuroinvasive disease case (95% CI 112–401). An estimate of 353 infections per each reported neuroinvasive disease case (95% CI 190–516) was obtained by analyzing the North Dakota data separately, which had 94 reported neuroinvasive cases among an estimated 33,000 infections (5.2% peak IgM prevalence × the state population of 633,837).

### Sensitivity Analyses

Two potential biases may have affected our estimated ratio of 256 WNV incident infections per reported neuroinvasive disease case. First, neuroinvasive cases may be underreported to ArboNET. A 20% underreporting of neuroinvasive cases to ArboNET alters the ratio to 205 WNV incident infections per reported neuroinvasive disease case (95% CI 90–320). Second, blood donors may underrepresent infections in the general population because prospective donors who are WNV-infected may self-defer or be deferred from donating. If the number of infections in the general population was underestimated by 20%, the ratio of WNV incident infections per reported neuroinvasive disease case would be 320 (95% CI 140–501).

## Discussion

This project, which collected and analyzed WNV screening data for 95% of US blood donations during the 2003 epidemic, identified 944 viremic donations among 4.6 million donations screened from July 1 to October 31. The number of viremic donors identified in 2003 is a slight underestimate since viremic donors identified by participating ABC and ARC centers outside the study time frame and viremic donors identified at nonparticipating collection centers and the military blood program were not included. The ABC and ARC data indicate that ≈1,000 West Nile viremic donors were identified in the United States in 2003 by prospective NAT screening, and consequently ≈1,500 potentially infectious blood components were interdicted before transfusion (*24*). This yield is particularly remarkable when compared with NAT screening for HIV-1 and HCV, which identified only 12 HIV and 170 HCV-infected antibody-negative donations among ≈39 million donations screened in the first 4 years of testing (*13*).

One goal of this project was to monitor the geographic and temporal distribution of WNV in the US blood donor population. We documented rates of viremic blood donors exceeding 3 per 1,000 donations in some states during the peak of the 2003 epidemic. The proportion of confirmed positive donations identified by minipool-NAT paralleled the neuroinvasive case reports in each state. Blood donor NAT screening data are useful for population surveillance because the testing has a rapid turnaround time, infections are identified soon after WNV acquisition, many of these infections remain asymptomatic, and typically those in whom symptoms develop are identified before illness onset. Communication of WNV donor screening data from blood centers to state and county health departments thus augments national surveillance and facilitates more complete national reporting of human WNV infections to CDC's ArboNET program (*24*).

Our estimate of an average 6.9-day period of viremia detectable by minipool-NAT correlates well with the duration of viremia that was documented after intentional WNV inoculation of human cancer patients in the 1950s (*25*). In those studies, the duration of viremia (detected by intracerebral inoculation in mice, which is less sensitive than minipool-NAT) correlated with underlying disease severity and averaged 6.2 days in a subset of relatively healthy patients.

Our results have limitations. We assumed that WNV incidence in blood donors reflects incidence in the general population. Blood donors differ from the general population with respect to age; however, serologic surveys indicate that age is not associated with the likelihood of WNV infection acquisition but is associated with severity of disease (*1**,**2**,**6**,**7*). Some racial, ethnic, and socioeconomic groups are also underrepresented in the blood donor population. Because WNV is a mosquitoborne arbovirus, incidence may vary among these demographic subgroups, which could bias extrapolations based on donor data. Moreover, potential donors with fever or headache are deferred from donation because the combined symptoms may indicate WNV infection; thus, blood donor screening data would underestimate infection incidence in the general population. However, we believe an underestimate is unlikely since the primary viremia phase of infection detected by minipool-NAT tends to precede development of WNV-related symptoms (*1**,**10**,**26*).

Although projections of seasonal incidence estimates based on donor data have limitations, they represent a source of data independent from national disease reporting. Completeness of reporting of WNV neuroinvasive cases to ArboNET is unknown and likely varies among states. The ratio of total infections to neuroinvasive cases is also not precisely known, thus adding uncertainty to incidence data extrapolated from such cases. Using blood donor screening data, we project that ≈256 people are infected with WNV for each person in whom neuroinvasive disease develops (95% CI 112–401). This ratio is similar to that observed in a serologic survey in Romania, which estimated that 1 in 140–320 infections results in neuroinvasive disease (*6*). Previous estimates of the total number of persons infected in the United States are based on a serologic survey in New York City that indicated that 1 in 140 infections (95% CI 61–217) results in neuroinvasive disease (*2**,**7*). Although CIs around the New York City estimate and our ratio overlap, the blood donor screening data suggest that previous projections may have underestimated the total number of persons infected. Similar analyses to determine the proportion of infections that result in febrile illness or other clinical manifestation of WNV would be of interest. However, reporting of these illnesses to ArboNet is incomplete and highly variable by state and over time and hence not appropriate for this purpose. Follow-up studies of viremic donors have demonstrated that febrile syndromes develop in 20% to 30% of patients (*26*), consistent with reports from other studies (*1**–**3*).

Our approach of using NAT yield data to project WNV infections has advantages over serologic strategies. Performing large-scale, community-based serologic surveys to estimate infection incidence is prohibitively expensive, is subject to participation bias, and can be biased by previous exposures to WNV or infections by other flaviviruses that cross-react on WNV IgM and IgG assays (*9**,**27**–**31*). Given the extent of recent WNV spread in the United States, interpretation of future serologic surveys will require determination of baseline prevalence before each epidemic year, evaluation of serial samples throughout the epidemic to accurately estimate infection incidence, or both.

In conclusion, our study demonstrates that in addition to preventing many transfusion-transmitted WNV infections, routine donor NAT screening has valuable public health applications, both as an early indicator of human epidemic activity regionally and as a surveillance tool to help monitor national infection incidence. In addition, this study highlights the value of establishing a national system for compiling blood donor data, which would enable ongoing and timely surveillance of WNV and other established and emerging infectious diseases.
